# Disorder‐specific and transdiagnostic vulnerability to posttraumatic stress symptoms: A machine learning approach

**DOI:** 10.1002/jts.70020

**Published:** 2025-11-10

**Authors:** Robert E. Fite, Johanna Thompson‐Hollands, John F. Buss, Lillian G. Lacy, Lorenzo Lorenzo‐Luaces, Lauren A. Rutter

**Affiliations:** ^1^ Department of Psychological and Brain Sciences Indiana University–Bloomington Bloomington Indiana USA; ^2^ Behavioral Science Division, National Center for PTSD at the VA Boston Healthcare System Boston Massachusetts USA; ^3^ Department of Psychiatry Boston University Chobanian & Avedisian School of Medicine Boston Massachusetts USA

## Abstract

A wide range of biological, cognitive, affective, and behavioral risk factors have been studied in relation to posttraumatic stress disorder. Previous work has often isolated a single risk factor or a small number of risk factors, making it is difficult to know which may be the most important to study or target in interventions. We used a supervised machine learning technique, *elastic net*, to test the associations between posttraumatic stress symptoms (PTSS) and several self‐reported risk factors at the full‐scale, subscale, and item levels in a large online sample (*N* = 1,186) of individuals who endorsed experiencing a *DSM‐5* Criterion A traumatic event, allowing for a broader and more granular understanding of the associations between transdiagnostic risk factors and PTSS. In our full‐scale model, posttraumatic cognitions, β = .28; anxiety sensitivity, β = .21; and posttraumatic maladaptive beliefs, β = .18, explained the largest amount of variance in PTSS. At the subscale level, heightened threat perceptions of harm, β = .30; negative cognitions about the self, β = .23; and cognitive sensitivity, β = .14, explained the largest amount of variance in PTSS. Meanwhile, at the item level, not feeling safe, not knowing oneself, and self‐blame for a traumatic event had the highest importance ratings. The identified variables may be important targets in future longitudinal and treatment research.

Posttraumatic stress disorder (PTSD) can be a debilitating condition with varying prevalence rates across different populations (Schein et al., 2021) and an estimated lifetime prevalence of approximately 7%–8% for the general population in the United States (Kessler et al., 2005, 2012). PTSD is defined by the experience of a Criterion A traumatic event (e.g., sexual assault, combat trauma), as outlined in the *Diagnostic and Statistical Manual of Mental Disorders* (5th ed., text rev.; *DSM‐5‐TR*; American Psychiatric Association [APA], 2022), coupled with trauma‐related intrusions, avoidance, changes in cognition or affect, and arousal or reactivity, as well as impaired functioning or distress that started or worsened following the traumatic event. A number of demographic characteristics (e.g., lower income, female sex) and aspects of the traumatic experience (e.g., military sexual trauma, childhood sexual abuse) have been identified as risk factors for developing PTSD (Klingensmith et al., 2014; Nöthling et al., 2017; Schein et al., 2021). However, there are gaps in knowledge regarding PTSD risk factors. The identification of PTSD risk factors is critical for prevention, treatment staging, and other clinical questions (e.g., treatment‐matching; Lynch et al., 2021; Nolen‐Hoeksema & Watkins, 2011; Rodriguez‐Seijas et al., 2015). Although some risk factors for PTSD may be relatively fixed (e.g., age, race), others represent mutable characteristics (e.g., cognitive biases). Both categories of risk factors may be useful in identifying individuals who should be prioritized for preventative or indicated interventions.

A common problem in studying risk factors is knowing which risk factors to consider in research and clinical work when so many potential and similar predictors exist (Semcho et al., 2023). Risk factors can be separated into those that are thought to be more specific to a particular condition (e.g., PTSD‐specific risk factors) and those that cut across diagnoses (i.e., transdiagnostic risk factors). PTSD shares many features with mood, anxiety, and related disorders, also known as *emotional disorders*, most notably overlapping symptoms (e.g., repetitive negative thinking) and effective treatments (e.g., cognitive restructuring, exposure). Indeed, PTSD was historically classified as an anxiety disorder. The most recent iterations of the *DSM* have placed it within a new category of trauma‐ and stressor‐related disorders (APA, 1980; Zoellner et al., 2011). Semcho et al. (2023) noted that many transdiagnostic constructs (e.g., experiential avoidance, intolerance of uncertainty) related to emotional disorders may be best conceptualized as “aversive reactivity to emotions,” which can be defined as the belief that certain emotions are undesirable, have negative consequences, and cannot be tolerated. Aversive reactivity to emotions has been proposed as a relevant construct to understanding emotional disorders broadly. In PTSD, for example, aversive reactivity may manifest in attempts to suppress thoughts and the avoidance of triggers.

Some risk factors can be considered more disorder‐specific. Perhaps the most specific risk factors for PTSD are posttraumatic cognitions. Cognitive theories of PTSD posit that certain beliefs (e.g., “The world is a dangerous place”) increase that individual's risk for developing and sustaining symptoms (Foa et al., 1989; LoSavio et al., 2017). These beliefs are positively associated with symptoms and may predict worse responses to treatment in some instances (Holder et al., 2019). Posttraumatic cognitions, including negative cognitions about the self, negative cognitions about the world, and self‐blame, have been the most studied beliefs in PTSD (Gómez de La Cuesta et al., 2019). Nonetheless, other beliefs have also been examined. For instance, Vogt et al. (2012) developed the Posttraumatic Maladaptive Beliefs Scale (PMBS), which measures beliefs focused on the present (e.g., the trustworthiness of others) versus trauma‐related beliefs.

Most PTSD investigations have studied risk factors in isolation, either focusing on potential transdiagnostic factors or more disorder‐specific risk factors. Because purported risk factors for PTSD may overlap in their definition and measurement, it would be ideal to simultaneously explore potential risk factors concurrently. Additionally, because it is unlikely that only one risk factor contributes to the risk of developing PTSD, understanding the relative impact, or combined impact, of risk factors is necessary. Unfortunately, many statistical approaches are ill‐equipped for handling a high volume of predictors that often overlap. Some work has applied machine learning approaches to identify multiple predictors of posttraumatic stress symptoms (PTSS; Christ et al., 2021; Papini et al., 2023). Machine learning is an umbrella term for statistical approaches that learn from data to facilitate prediction efforts. Some machine learning methods have been developed for use in situations in which there are a large number of predictors, such as in predicting the risk for PTSD, and it may be of interest to identify a small subset of risk factors (e.g., Christ et al., 2021).

We investigated the relative importance of both transdiagnostic risk factors relating to aversive reactivity to emotions (i.e., intolerance of uncertainty, experiential avoidance, anxiety sensitivity, expressive suppression, contrast avoidance) and those that may be more disorder‐specific (i.e., posttraumatic cognitions, posttraumatic maladaptive beliefs). Due to the high number of variables we were interested in investigating, as well as the large amount of overlap between variables, we used *elastic net*, a supervised machine learning technique that is well‐equipped for handling a high volume of predictor variables, including variables that may have high multicollinearity (Zou & Hastie, 2005). A benefit of using elastic net over other machine learning techniques or traditional ordinary least squares regression is the ability to handle multidimensional data while retaining a linear model that is easily interpretable. This approach allowed us to study a range of overlapping risk factors to identify which are most strongly associated with PTSS. Ours aims are consistent with calls for investigating transdiagnostic risk factors in the study of traumatic stress using statistical approaches like machine learning (Adams et al., 2024). In addition to examining a full‐scale model and a model that included scales and subscales (scale/subscale model), we examined the association between single items and PTSS. In psychology, traditional research on self‐reported risk factors is typically conducted using multi‐item questionnaires, which are often recommended to measure key constructs due to the added reliability they provide. Of note, administering full survey scales may not be feasible in many settings, underscoring the need to identify briefer assessments (e.g., single items). Past work has found evidence of similar validity of single‐item measures versus multi‐item measures of mental health (Ahmad et al., 2014; Song et al., 2023). In some cases, single‐item measures may even be preferable to multi‐item measures, as single‐item measures allow for a more granular understanding of constructs that could inform idiographic models of psychopathology (Allen et al., 2022; Song et al., 2023). Additionally, identifying the most important items could have implications for ecological momentary assessment and treatment studies in which collecting full scales may not always be feasible (Allen et al., 2022; Song et al., 2023).

## METHOD

### Participants and procedure

All study procedures were approved by the Indiana University Institutional Review Board. Participants (*N* = 1,581) were recruited from Prolific, an online research participant recruitment platform. The study was cross‐sectional, and participants responded to a range of demographic questions and self‐report surveys through the Qualtrics survey platform. Participants were compensated $4 (USD) for their time, which was estimated to be 20 min in length. To be included in the final analysis, participants had to complete more than 60% of the study, meet at least two of the three data quality inclusion criteria, and endorse experiencing a *DSM‐5* Criterion A traumatic event (assessed via self‐report on the PTSD Checklist for *DSM‐5* with Criterion A; PCL‐5; Weathers et al., 2013). The data quality inclusion criteria were used to protect against potential fraudulent responders (e.g., “bots”) and rapid or inattentive responding. The criteria consisted of being in the upper 95th percentile of completion time, having a reCAPTCHA score of greater than or equal to 0.5, and having a relevant ID fraud score of less than 31. Using participant metadata (e.g., operating system, screen resolution), Qualtrics produces reCAPTCHA scores and relevant ID fraud scores and suggests these cutoffs for differentiating between human and bot respondents (Qualtrics, n.d.); these cutoffs have been used in previous research for bot detection (Lorenzo‐Luaces & Howard, 2023). A total of 121 participants did not meet the data quality inclusion criteria and were removed. An additional 274 participants were removed because they denied exposure to a Criterion A traumatic event. These steps resulted in a final sample of 1,186 participants (see Table 1).

**TABLE 1:** Participant characteristics

Variable	*M*	*SD*
Age (years)	42.48	13.50
PCL‐5 score	18.48	18.07

	***n***	**%**
Race/ethnicity		
American Indian or Alaska Native	32	2.7
Asian	88	7.4
African/Black	168	14.2
Native Hawaiian or Pacific Islander	3	0.3
European/White	941	79.3
Hispanic/Latino	111	9.4
Sex assigned at birth		
Female	682	57.5
Male	498	42.0
Gender		
Female	651	54.9
Male	493	41.6
Transgender or nonbinary	39	3.3
Sexual orientation		
Straight/heterosexual	937	79.0
Lesbian/gay	54	4.6
Bisexual/pansexual	155	13.1
Other	31	2.6
Past PTSD diagnosis		
Yes	215	18.1
No	876	73.9
I don't know	16	1.4
No, but I should be	79	6.7
Above PCL‐5 probable PTSD cutoff^a^	254	21.4

*Note*: *N* = 1,186. All participants endorsed Criterion A traumatic event exposure per the *Diagnostic and Statistical Manual of Mental Disorders* (5th ed.; *DSM‐5*). PCL‐5 = PTSD Checklist for *DSM‐5*; PTSD = posttraumatic stress disorder.

^a^ 33 or higher.

### Measures

Omega values are reported as a measure of internal consistency for each scale or subscale. Omega values are more optimal in assessing internal consistency when the assumptions of tau‐equivalence are violated (e.g., equal item means, equal item variance; Dunn et al., 2014).

#### PTSS

As a measure of PTSS, participants completed the PCL‐5 (Weathers et al., 2013), which consists of 20 items reflecting *DSM‐5* PTSD symptoms (APA, 2013). After reading a description of traumatic events, participants were asked to “briefly identify the worst event” they had experienced. They were then asked to indicate how much they were bothered by each symptom (e.g., intrusive memories, hyperarousal) over the past month on a scale from 0 (*not at all*) to 4 (*extremely*), with scores summed (range: 0–80) and higher scores indicating higher symptom levels. Suggested cutoff scores for a probable PTSD diagnosis range from 31 to 33 (Bovin et al., 2016). For the purposes of characterizing our sample, we used a cutoff score of 33. The PCL‐5 has shown good psychometric properties (Blevins et al., 2015), and in the current study, the PCL‐5 demonstrated excellent internal consistency, ω = .97.

##### Negative posttraumatic cognitions

The Posttraumatic Cognitions Inventory‐9 (PTCI‐9; Wells et al., 2019) is a shortened version of the PTCI (Foa et al., 1999) and appears to have more robust psychometric properties than the original measure (Byllesby et al., 2022). The PTCI‐9 has three subscales measuring (a) negative cognitions about the self (PTCI‐NC‐Self; e.g., “I feel like I don't know myself anymore.”), (b) negative cognitions about the world (PTCI‐NC‐World; e.g., “People can't be trusted.”), and (c) self‐blame (e.g., “The event happened because of the way I acted.”). Participants rated items on a scale of 1 (*totally disagree*) to 7 (*totally agree*), with scores summed and higher scores indicating higher levels of negative posttraumatic cognitions. In the current study, internal consistency was good for the NC‐Self, ω = .88; NC‐World, ω = .90; and Self‐Blame subscales, ω = .81.

##### Posttraumatic maladaptive beliefs

The PMBS (Vogt et al., 2012) consists of 15 items and has three subscales: Threat of Harm (PMBS‐Threat; e.g., “I don't feel safe anywhere anymore”), Self‐Worth and Judgement (PMBS‐Self‐Worth; e.g., “I trust my own judgment” [reverse scored]), and Reliability and Trustworthiness of Others (PMBS‐Trust; e.g., “Some people can be trusted” [reverse scored]). Respondents rated items on a scale of 1 (*not true*) to 7 (*completely true*), and scores were summed, with higher scores indicating higher levels of maladaptive beliefs. The PMBS has shown good psychometric properties, and in the current study, internal consistency was good for the Threat, ω = .85; Self‐Worth, ω = .84; and Trust subscales, ω = .86.

##### Intolerance of uncertainty

The Intolerance of Uncertainty Scale‐Short (IUS‐12; Carleton et al., 2007) has two subscales: Prospective Anxiety (IUS‐Prospective) and Inhibitory Anxiety (IUS‐Inhibitory). Prospective anxiety refers to the anxiety or negative emotions that are caused by uncertainty (e.g., “Unforeseen events upset me greatly”), whereas inhibitory anxiety refers to the avoidance or disruption of behaviors caused by uncertainty (e.g., “I must get away from all uncertain situations”). Participants rated items on a scale of 1 (*not at all characteristic*) to 5 (*entirely characteristic*), with scores summed and higher scores indicating higher levels of intolerance of uncertainty. The IUS‐12 has demonstrated good psychometric properties. In the current study, internal consistency was excellent for both the Prospective, ω = .90, and Inhibitory subscales, ω = .93.

##### Experiential avoidance

The Behavioral Experiential Avoidance Questionnaire (BEAQ; Gámez et al., 2014) is a 15‐item measure of experiential avoidance. Participants responded to items (e.g., “The key to a good life is never feeling any pain”) on a scale of 1 (*strongly disagree*) to 6 (*strongly agree*), with scores summed and higher scores indicating higher levels of experiential avoidance. The BEAQ has demonstrated good psychometric properties, and internal consistency was excellent in the current study, ω = .91.

##### Anxiety sensitivity

The Anxiety Sensitivity Index–3 (ASI‐3; Taylor et al., 2007) is an 18‐item measure that consists of three subscales: Physical Sensitivity (ASI‐Physical; e.g., “It scares me when my heart beats rapidly”), Cognitive Sensitivity (ASI‐Cognitive; e.g., “When I cannot keep my mind on a task, I worry that I might be going crazy”), and Social Sensitivity (ASI‐Social; e.g., “I worry that other people will notice my anxiety”). Participants rated items on a scale of 0 (*very little*) to 4 (*very much*), with scores summed and higher scores indicating higher levels of anxiety sensitivity. The ASI‐3 has demonstrated good psychometric properties (Wheaton et al., 2012), and in the current study, showed excellent internal consistency for the Physical, ω = .91, and Cognitive subscales, ω = .92, and good internal consistency for the Social subscale, ω = .84.

##### Emotion regulation

The 10‐item Emotion Regulation Questionnaire (ERQ; Gross & John, 2003) includes two subscales that measure two types of emotion regulation strategies: Cognitive Reappraisal (ERQ‐Reappraisal; e.g., “When I want to feel less negative emotion, I change the way I'm thinking about the situation”) and Expressive Suppression (ERQ‐Suppression; e.g., “When I am feeling negative emotions, I make sure not to express them”). Participants rated items on a scale of 1 (*strongly disagree*) to 7 (*strongly agree*), with scores summed and higher scores indicating more use of the emotion regulation strategy. The ERQ has demonstrated evidence of good psychometric properties (Preece et al., 2021), and in the current study, internal consistency was excellent for the Reappraisal subscale, ω = .93, and good for the Suppression subscale, ω = .85.

##### Contrast avoidance

The Contrast Avoidance Questionnaire‐General Emotion (CAQ‐GE; Llera & Newman, 2017) is a 25‐item measure that assesses two components of contrast avoidance: discomfort as a result of emotional shifts (CAQ‐Discomfort; e.g., “I am particularly uneasy with sharp shifts in my negative emotion”) and creating and maintaining negative emotions as a means of avoiding shifts in emotions (CAQ‐Creating; e.g., “I focus on the negative because I want to be emotionally prepared in case something terrible happens”). Participants rated items on a scale of 1 (*never*) to 5 (*always*), with scores summed and higher scores indicating higher levels of contrast avoidance. The CAQ‐GE has demonstrated good psychometric properties, and in the current study, internal consistency was excellent for both the Discomfort, ω = .91, and Creating subscales, ω = .97.

### Data analysis

All analyses were conducted in R (Version 4.3.1). When conducting analyses with a high number of parameters, common statistical approaches, such as ordinary least squares regression, are prone to overfitting, which leads to overestimated coefficients and poor replicability (Babyak, 2004). Several supervised learning approaches (i.e., using labeled data that are split into separate training and testing datasets to predict or classify variables) have been developed to reduce overfitting and handle highly correlated parameters. Least absolute shrinkage and selection operator (LASSO) regression applies a penalty (λ) that will shrink the size of coefficients, including, if they are small enough, to 0 (Tibshirani, 1996). LASSO regression will remove parameters that are of low or no importance from the model, therefore functioning as a variable selection method. Ridge regression (Hoerl & Kennard, 1970) also shrinks the size of coefficients but does not lower them to 0 (Ogutu et al., 2012). Elastic net regression, which the current study employed, combines the regularization penalties of LASSO regression and ridge regression. Two hyperparameters are used in elastic net regression: alpha (α) and lambda (λ). The alpha value varies between 0 and 1. When alpha is 0, the result is a ridge regression, and when alpha is 1, the result is a LASSO regression; thus, when alpha is greater than 0 but less than 1, elastic net regression is performed.

Among participants who met the inclusion criteria, missing data were low for each variable included in our models (< 0.001%). To increase power and reduce bias, missing data were handled using random‐forest single imputation with the *missForest* package in R (Stekhoven, 2022; Stekhoven & Buehlmann, 2012). Random forest was used with 500 trees and 100 iterations. All variables were standardized for interpretability. We trained and tested three separate elastic net models using *glmnet* via the *caret* package in R (Friedman et al., 2010; Kuhn, 2008). The first model examined the association between potential risk factors at the full‐scale level and PTSS (i.e., full‐scale model). The second model examined the associations between subscales assessing the potential risk factors, in the case of the BEAQ, the full scale, and PTSS (i.e., scale/subscale model). The third model examined the associations between individual items from the scales and PTSS. We also analyzed these models with demographic variables included; the descriptions of these analyses and results can be found in the Supplementary Materials and Supplementary Figures S1–S3. We used a training–testing split of 70/30, which has been commonly used in prior research (Christ et al., 2021). We used 10‐fold cross‐validation on the training data and employed a grid search to select the alpha and lambda values. The training models were used to determine the best alpha value, as well as the lambda within 1 standard error of the minimum cross‐validation error. We then calculated importance ratings, which were scaled to the largest coefficient in the model. Finally, we used the testing data to quantify model performance using the hyperparameter penalties from the training data. Model fit was examined using root mean squared error (RMSE), mean absolute error (MAE), and *R* ^2^ values. RMSE and MAE are both absolute fit statistics. Lower RMSE and MAE values indicate better model fit, and *R* ^2^ values reflect the amount of variance explained in the model.

## RESULTS

See Supplementary Table S1 for means and standard deviations for all variables, as well as bivariate correlations between variables. As shown, most risk factor variables demonstrated moderate‐to‐large positive associations with PTSS.

### Elastic net regression

#### Full‐scale model

**FIGURE 1:**
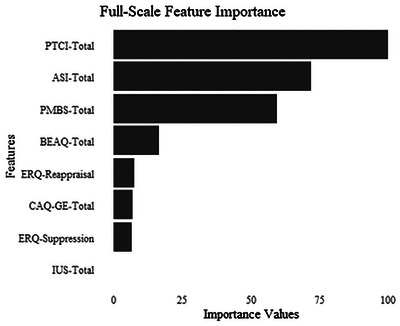
Importance values for full scales relative to posttraumatic stress symptoms in the training model *Note*: PTCI = Posttraumatic Cognitions Inventory; ASI = Anxiety Sensitivity Index; PMBS = Posttraumatic Maladaptive Beliefs Scale; BEAQ = Behavioral Experiential Avoidance Questionnaire; ERQ = Emotion Regulation Questionnaire; CAQ = Contrast Avoidance Questionnaire–General Emotion; IUS = Intolerance of Uncertainty Scale–Short.

We conducted an elastic net regression to examine the association between the full‐scale risk factors and PTSS. The ERQ does not yield a total score, so we included the two subscales in the model. As a result of the cross‐validation, optimal penalties were selected (α = 0, λ = .15) to calculate the coefficients of the parameters. The alpha value was 0, thus, a ridge regression was performed. See Figure 1 for the importance ratings of the full scales from the training model and Table 2 for the coefficients from the training model. As shown, the PTCI‐9, ASI, and PMBS had the highest importance ratings, with the BEAQ, ERQ‐Reappraisal, CAQ‐GE, and ERQ‐Suppression scales/subscales included in the model (listed in order of importance). Only the IUS‐12 was not included in the model. There appeared to be good model fit for the training data, RMSE = .6990, MAE = .5339, and the testing data. Model characteristics can be found in Table 3.

**TABLE 2:** Coefficients for the full‐scale elastic net training model

Scale	β
(Intercept)	.0000
PTCI total	.2784
PMBS total	.1846
BEAQ total	.0794
ASI total	.2083
ERQ‐Reappraisal	.0456
ERQ‐Suppression	−.0370
CAQ‐GE total	.0602
IUS total	.0422

*Note*: PTCI = Posttraumatic Cognitions Inventory; PMBS = Posttraumatic Maladaptive Beliefs Scale; BEAQ = Behavioral Experiential Avoidance Questionnaire; ASI = Anxiety Sensitivity Index–3. ERQ = Emotion Regulation Questionnaire; CAQ‐GE = Contrast Avoidance Questionnaire–General Emotion; IUS = Intolerance of Uncertainty Scale–Short.

**TABLE 3:** Model performance of elastic net regressions

Model	RMSE	MAE	*R* ^2^
Full‐scale training	.6990394	.5339958	.5110683
Full‐scale test	.6799752	.5239742	.5390912
Scale/subscale training	.6696682	.5043295	.5515287
Scale/subscale test	.6483775	.5023802	.5800603
Item‐level training	.6427613	.4847132	.5890734
Item‐level test	.6371765	.4883283	.5947256

*Note*: RMSE = root mean square error; MAE = mean absolute error.

#### Scale/subscale model

**FIGURE 2:**
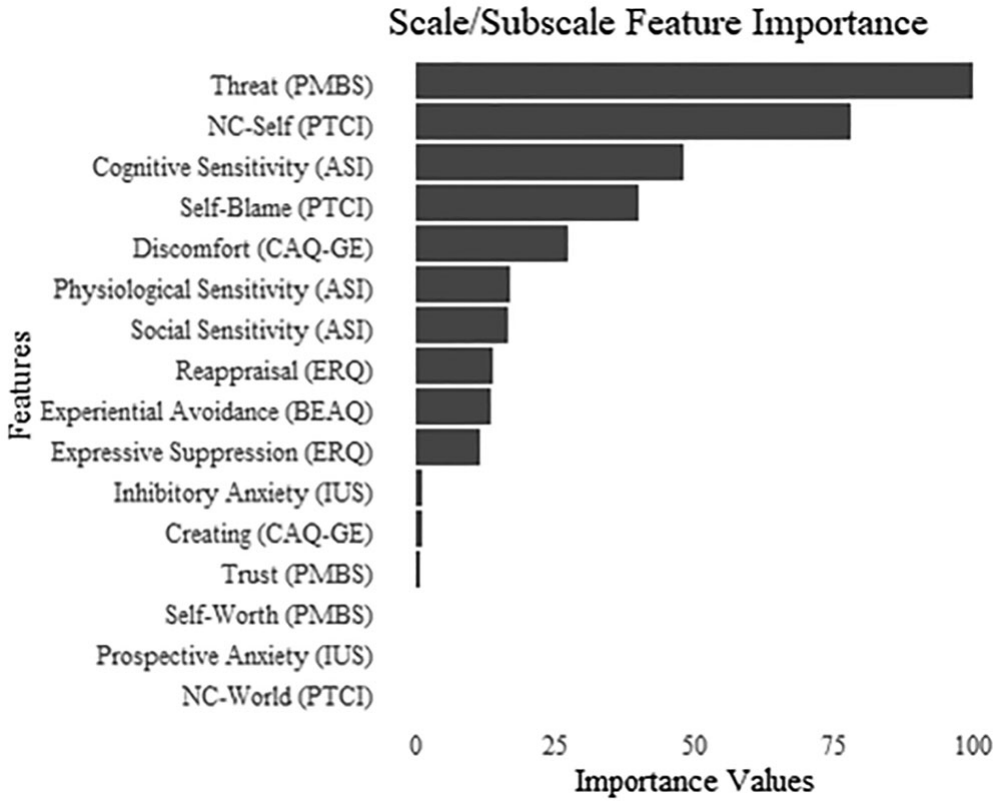
Importance values for each scale/subscale relative to posttraumatic stress symptoms in the training model *Note*: PMBS = Posttraumatic Maladaptive Beliefs Scale; PTCI = Posttraumatic Cognitions Inventory; NC = negative cognitions; ASI = Anxiety Sensitivity Index; CAQ = Contrast Avoidance Questionnaire–General Emotion; BEAQ = Behavioral Experiential Avoidance Questionnaire; ERQ = Emotion Regulation Questionnaire; IUS = Intolerance of Uncertainty Scale–Short.

We conducted an elastic net regression with scales/subscales entered as parameters in the model. Optimal penalties (α = .1, λ = .04) were selected. Given the alpha value, many of the variables were included in the model. The PMBS‐Threat, PTCI‐NC‐Self, and ASI‐Cognitive subscales had the largest coefficients. See Table 4 for the coefficients for the training model. Figure 2 shows the importance ratings for the risk factors from the training model. The elastic net model appeared to fit the training data well, RMSE = .6698, MAE = 0.5045, and generalized to the testing data (i.e., slightly lower RMSE and MAE values).

**TABLE 4:** Coefficients for the scale/subscale elastic net training model

Scale/subscale	β
(Intercept)	.0000
PTCI‐NC‐Self	.2331
PTCI‐NC‐World	−.0007
PTCI‐Self‐Blame	.1166
PMBS‐Threat	.3015
PMBS‐Self‐Worth	.0000
PMBS‐Trust	−.0094
BEAQ total	.0379
ASI‐Physical	.0467
ASI‐Cognitive	.1420
ASI‐Social	.0483
ERQ‐Reappraisal	.0425
ERQ‐Suppression	−.0370
CAQ‐Creating	‐.0129
CAQ‐Discomfort	.0839
IUS‐Prospective	.0000
IUS‐Inhibitory	.0021

*Note*: PTCI = Posttraumatic Cognitions Inventory; NC = negative cognitions; PMBS = Posttraumatic Maladaptive Beliefs Scale; BEAQ = Behavioral Experiential Avoidance Questionnaire; ASI = Anxiety Sensitivity Index; ERQ = Emotion Regulation Questionnaire; CAQ = Contrast Avoidance Questionnaire–General Emotion; IUS = Intolerance of Uncertainty Scale–Short.

#### Item‐level model

**FIGURE 3:**
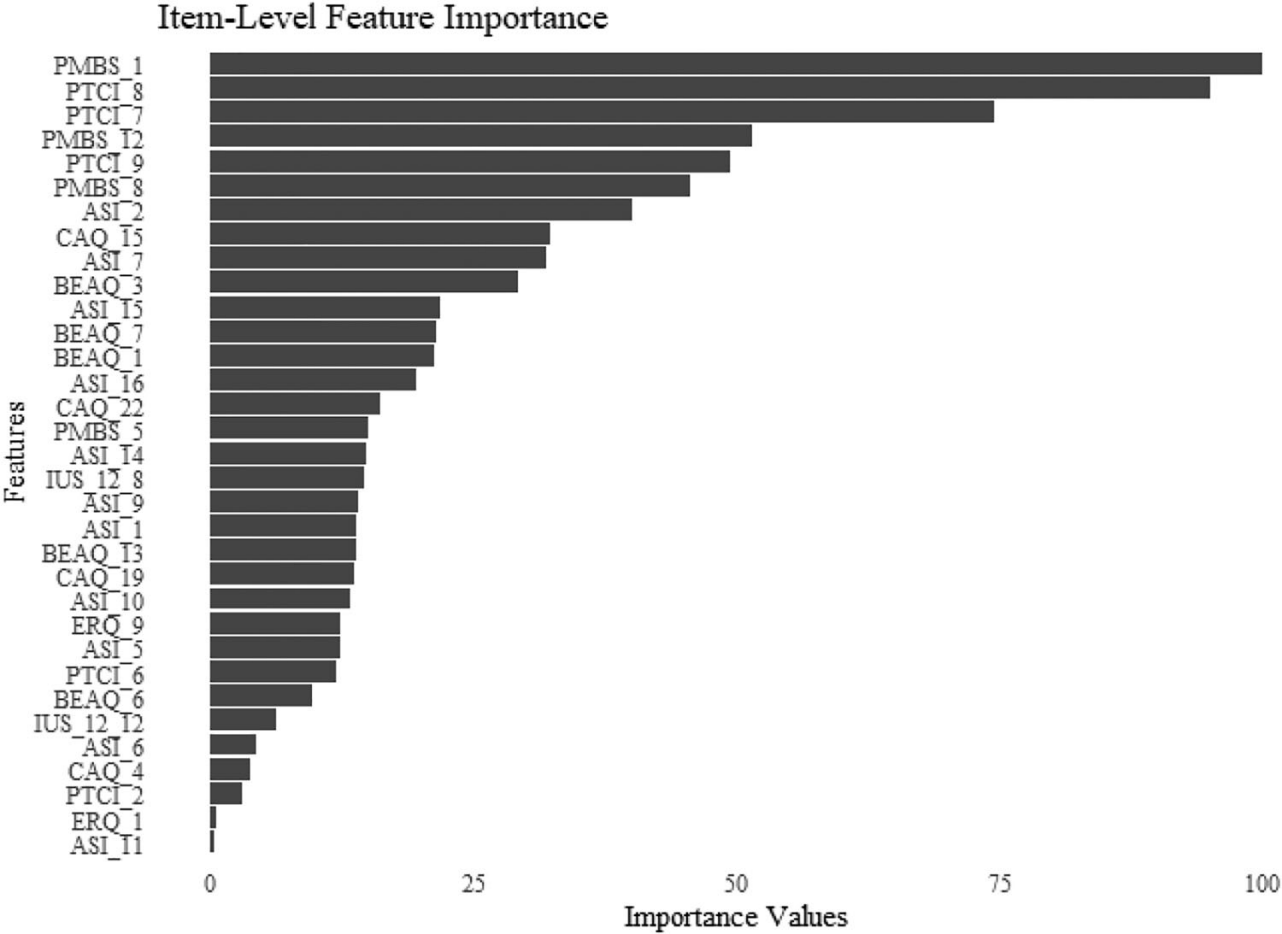
Importance values for scale items relative to posttraumatic stress symptoms in the training model *Note*: Items without a scaled importance larger than zero are not included. PMBS = Posttraumatic Maladaptive Beliefs Scale; PTCI = Posttraumatic Cognitions Inventory; ASI = Anxiety Sensitivity Index; CAQ = Contrast Avoidance Questionnaire–General Emotion; BEAQ = Behavioral Experiential Avoidance Questionnaire; IUS = Intolerance of Uncertainty Scale–Short; ERQ = Emotion Regulation Questionnaire.

To gain a more granular understanding of these associations, we conducted an elastic net regression to examine the association between all individual scale items and PTSS. Optimal penalties (α = .2, λ = .08) were included to calculate the coefficients of the parameters. See Figure 3 for the importance ratings of the training model when individual items were entered as parameters. Coefficients from the training model can be found in Supplementary Table S2. As shown, the items with the highest importance ratings were from the PTCI‐9 and PMBS. Finally, we examined how well our model performed with the training data, RMSE = .6428, MAE = .4847, which generalized well to the test data. The analyses revealed that the models performed quite similarly in terms of performance metrics.

## DISCUSSION

We examined the associations between transdiagnostic and disorder‐specific risk factors and PTSS. When we examined total scales, three measures stood out from the rest: the PTCI‐9, ASI‐3, and PMBS. A heightened sense of threat was the most important feature in our scale/subscale model. This finding aligns with theory and research focusing on perceptions of threat in the context of PTSD (Huskey et al., 2022; Kredlow et al., 2022). Recent work has helped clarify the link between PTSD symptoms and difficulty in updating cognitions about danger when disconfirmatory information is received (Haim‐Nachum et al., 2024; Sopp et al., 2022). This research suggests that the process may not be solely centered on initial appraisals but also on how safety cues are or are not used to update appraisals. At the item level, cognitions that had the highest importance in our model reflected threat of harm, negative cognitions about the self, and self‐blame. These results highlight differences that emerge depending upon whether full scales, subscales, or individual items are used.

Among the risk factors included in our analyses, only items from the PTCI‐9 Self‐Blame subscale explicitly referred to a specific event (i.e., the traumatic event). Interestingly, there were three features that had higher importance that were not event‐focused (i.e., threat of harm, negative cognitions about the self, and cognitive sensitivity). Although we are not able to infer a causal direction of this association, it is worthwhile to consider how these findings may relate to developmental models of psychopathology. Staging models posit that at early stages of psychopathology development, general risk factors are present, but symptoms are either dormant or subthreshold (McGorry et al., 2018). The “triple vulnerability” model differentiates between biological vulnerability, generalized psychological vulnerability, and disorder‐specific vulnerability (Barlow, 2000). Heightened perceptions of threat, maladaptive cognitions about the self, and cognitive anxiety sensitivity, may broadly convey a risk for psychopathology but be especially relevant for PTSS following exposure to a traumatic or stressful event (Howard et al., 2024) or when more specific risk factors emerge (e.g., self‐blame). An important future direction for research will be to examine these risk factors longitudinally to better understand how they contribute to the development of PTSS and other internalizing symptoms.

A main rationale for studying these risk factors is that researchers and clinicians may be able to target these factors either preemptively (i.e., through preventative interventions) or after clinically significant symptoms have developed. Targeting transdiagnostic risk factors has been of much interest in the clinical literature (Lorenzo‐Luaces et al., 2024; Thompson‐Hollands et al., 2014); however, there are a plethora of potential transdiagnostic therapeutic targets, often with overlapping characteristics (Semcho et al., 2023). At the bivariate level, all potential risk factors were associated with PTSS, with effect sizes (*r* values) ranging from ‐.13 to .66. However, our analyses suggest that, when controlling for one another, only a handful of the variables were strongly associated with PTSS. Future research should explore the practical implications of these findings. Targeting distinct putative risk factors may result in superior treatment results (e.g., Craske et al., 2023, 2016). Our findings indicate heightened perceptions of threat and distorted cognitions about the self as potentially important therapeutic targets for PTSD, while also identifying other, more novel risk factors to target, such as discomfort related to emotional shifts.

The cross‐sectional design does not allow us to make causal claims about the directionality of these associations. Past research has examined some of these purported risk factors longitudinally in their associations with PTSS (Brown et al., 2019). However, more research will be needed to examine risk factors that appear relevant to PTSD based on these analyses (e.g., cognitive sensitivity, contrast avoidance) but for which temporal associations with PTSD are less clear or unknown. Another limitation of the current study was the reliance on self‐report measures of risk factors and symptoms. One possible explanation for the results is that the distinction between the measures of posttraumatic cognitions and symptoms is blurred. For instance, some researchers have written about the “jangle fallacy,” in which measures that are purportedly measuring distinct concepts are actually measuring the same thing (Flake & Fried, 2020). For this reason, it is valuable to also consider risk factors that may have had lower importance ratings but were retained in our models. For prediction efforts, at the level of a given individual, a variable with a seemingly low importance rating may still be a strong contributor to PTSD symptoms, such as if the individual has an exceptionally high score. In longitudinal research, variables with relatively lower importance may still prospectively predict PTSD severity. In mechanistic research, variables with seemingly low importance may still be causally related to PTSD symptom severity. There are also several strengths of this study, including a large sample size (*N* = 1,186), the use of regularization penalties to reduce the risk of overfitting models, and the examination of a wide range of transdiagnostic and disorder‐specific risk factors.

Our findings highlight the importance of studying both transdiagnostic and disorder‐specific risk factors. Additionally, our findings highlight the value of focusing on specific subscales within a broader construct. For example, the PTCI has three subscales: Negative Cognitions About the Self, Negative Cognitions About the World, and Self‐blame. Although the PTCI‐NC‐Self subscale was moderately associated with PTSS, the PTCI‐NC‐World subscale was not, and the PTCI‐Self‐Blame subscale demonstrated an association somewhere in between. Although we were focused on total PCL‐5 scores as our outcome variable, future work may focus on the unique association between these risk factors and specific symptom clusters.

As our findings suggest and in line with past research and reviews, a range of risk factors are relevant to PTSS (Brown et al., 2019; Rutter et al., 2022; Thompson‐Hollands et al., 2017). It will be useful for treatment studies to test the effects of interventions not only on commonly studied risk factors (e.g., posttraumatic cognitions) but also on more novel constructs related to aversive reactivity, such as cognitive sensitivity and contrast avoidance. It will also be important to examine how these risk factors may provide insights into precision treatment efforts. Analytical approaches that are well‐suited for investigating precision treatment questions often require sample sizes that exceed the number of participants in most randomized controlled trials. To overcome this obstacle, researchers may consider pooling data sets, relaxing inclusion criteria, or conducting remote trials that can often be done more efficiently (Lorenzo‐Luaces et al., 2018; Lorenzo‐Luaces & Fite, 2024). Alternatively, tightly controlled experimental studies aimed at reducing these risk factors may provide insight into potential novel therapeutics (Lorenzo‐Luaces, 2023).

## AUTHOR CONTRIBUTIONS

Robert E. Fite: Conceptualization; Writing ‐ original draft; Writing ‐ review & editing; Visualization; Formal analysis; Project administration. Johanna Thompson‐Hollands: Writing ‐ review & editing; Conceptualization. John F. Buss: Writing ‐ review & editing; Formal analysis. Lillian G. Lacy: Writing ‐ review & editing; Project administration. Lorenzo Lorenzo‐Luaces: Writing ‐ review & editing; Formal analysis; Conceptualization. Lauren A. Rutter: Writing ‐ review & editing; Conceptualization; Project administration.

## AUTHOR NOTE

The views expressed herein are solely those of the authors and do not reflect an endorsement by or the official policy or position of the U.S. Department of Veterans Affairs or the U.S. Government.

## OPEN PRACTICES STATEMENT

Code for the elastic net models is available on the Open Science Framework (https://osf.io/4cqk6/). The study reported in this article was not formally preregistered. Neither the data nor the materials have been made available on a permanent third‐party archive; requests for the data or materials should be sent via email to the lead author at refite@iu.edu.

## Supporting information



SUPPORTING INFORMATION
