# Supplementary material for: Disorder‐specific and transdiagnostic vulnerability to posttraumatic stress symptoms: A machine learning approach
Source: J Trauma Stress. 2025 Nov 10;39(1):86–97. doi: 10.1002/jts.70020 (PMC12890739; doi:10.1002/jts.70020)
Supplement: Supplementary file 1 — SUPPORTING INFORMATION [file JTS-39-86-s001.docx]

Supplementary Analyses

**Supplementary Table 1.***Means, standard deviations, and bivariate correlations between posttraumatic stress symptoms (PTSS) and potential PTSS risk factors*

| **Variable** | **1.** | **2.** | **3.** | **4.** | **5.** | **6.** | **7.** | **8.** | **9.** | **10.** | **11.** | **12.** | **13.** | **14.** | **15.** | **16.** | **17.** |
| --- | --- | --- | --- | --- | --- | --- | --- | --- | --- | --- | --- | --- | --- | --- | --- | --- | --- |
| 1. PCL-5 total | – | .60 ^***^ | .49 ^***^ | .41 ^***^ | .66 ^***^ | .48 ^***^ | .41 ^***^ | .49 ^***^ | -.13 ^***^ | .25 ^***^ | .50 ^***^ | .49 ^***^ | .38 ^***^ | .51 ^***^ | .46 ^***^ | .55 ^***^ | .46 ^***^ |
| 2. PTCI-NC-Self |  | – | .47 ^***^ | .46 ^***^ | .62 ^***^ | .70 ^***^ | .50 ^***^ | .44 ^***^ | -.29 ^***^ | .32 ^***^ | .58 ^***^ | .43 ^***^ | .30 ^***^ | .50 ^***^ | .35 ^***^ | .47 ^***^ | .36 ^***^ |
| 3. PTCI-NC-World |  |  | – | .36 ^***^ | .73 ^***^ | .37 ^***^ | .56 ^***^ | .43 ^***^ | -.10 ^**^ | .31 ^***^ | .42 ^***^ | .37 ^***^ | .32 ^***^ | .38 ^***^ | .32 ^***^ | .36 ^***^ | .35 ^***^ |
| 4. PTCI- Self-Blame |  |  |  | – | .37 ^***^ | .39 ^***^ | .25 ^***^ | .26 ^***^ | -.08 ^*^ | .22 ^***^ | .33 ^***^ | .23 ^***^ | .18 ^***^ | .31 ^***^ | .22 ^***^ | .29 ^***^ | .23 ^***^ |
| 5. PMBS-Threat |  |  |  |  | – | .54 ^***^ | .64 ^***^ | .57 ^***^ | -.19 ^***^ | .38 ^***^ | .59 ^***^ | .49 ^***^ | .46 ^***^ | .57 ^***^ | .43 ^***^ | .50 ^***^ | .47 ^***^ |
| 6. PMBS-Self-Worth |  |  |  |  |  | – | .57 ^***^ | .43 ^***^ | -.42 ^***^ | .32 ^***^ | .57 ^***^ | .49 ^***^ | .28 ^***^ | .54 ^***^ | .31 ^***^ | .45 ^***^ | .36 ^***^ |
| 7. PMBS-Trust |  |  |  |  |  |  | – | .35 ^***^ | -.27 ^***^ | .39 ^***^ | .50 ^***^ | .35 ^***^ | .28 ^***^ | .41 ^***^ | .22 ^***^ | .32 ^***^ | .28 ^***^ |
| 8. BEAQ total |  |  |  |  |  |  |  | – | -.11 ^**^ | .43 ^***^ | .58 ^***^ | .60 ^***^ | .55 ^***^ | .63 ^***^ | .48 ^***^ | .54 ^***^ | .52 ^***^ |
| 9. ERQ-Reappraisal |  |  |  |  |  |  |  |  | – | -.06 | -.31 ^***^ | -.18 ^***^ | -.11 ^**^ | -.25 ^***^ | -.08 ^*^ | -.16 ^***^ | -.06 |
| 10 ERQ-Suppression |  |  |  |  |  |  |  |  |  | – | .41 ^***^ | .27 ^***^ | .27 ^***^ | .31 ^***^ | .20 ^***^ | .28 ^***^ | .32 ^***^ |
| 11. CAQ- Creating |  |  |  |  |  |  |  |  |  |  | – | .65 ^***^ | .56 ^***^ | .67 ^***^ | .43 ^***^ | .56 ^***^ | .50 ^***^ |
| 12. CAQ-Discomfort |  |  |  |  |  |  |  |  |  |  |  | – | .58 ^***^ | .67 ^***^ | .45 ^***^ | .54 ^***^ | .54 ^***^ |
| 13. IUS- Prospective |  |  |  |  |  |  |  |  |  |  |  |  | – | .69 ^***^ | .43 ^***^ | .44 ^***^ | .52 ^***^ |
| 14. IUS-Inhibitory |  |  |  |  |  |  |  |  |  |  |  |  |  | – | .51 ^***^ | .59 ^***^ | .56 ^***^ |
| 15. ASI-Physical |  |  |  |  |  |  |  |  |  |  |  |  |  |  | – | .67 ^***^ | .60 ^***^ |
| 16. ASI-Cognitive |  |  |  |  |  |  |  |  |  |  |  |  |  |  |  | – | .64 ^***^ |
| 17. ASI-Social |  |  |  |  |  |  |  |  |  |  |  |  |  |  |  |  | – |
| *M* | 18.48 | 6.66 | 11.72 | 6.94 | 16.83 | 13.53 | 14.91 | 52.09 | 30.38 | 15.38 | 40.53 | 20.35 | 22.11 | 12.11 | 6.88 | 5.26 | 9.21 |
| *SD* | 18.07 | 4.62 | 5.25 | 4.49 | 6.93 | 6.33 | 6.55 | 13.05 | 7.50 | 5.87 | 17.13 | 6.24 | 6.68 | 5.87 | 6.38 | 6.04 | 5.89 |

*Note*: *N* = 1,186. All participants endorsed Criterion A traumatic event exposure per the *Diagnostic and Statistical Manual of Mental Disorders* (5th ed.; *DSM-5*). PCL-5 = PTSD Checklist for *DSM-5*; PTCI = Posttraumatic Cognitions Inventory–9; NC = negative cognitions; PMBS = Posttraumatic Maladaptive Beliefs Scale; BEAQ = Behavioral Experiential Avoidance Questionnaire; ERQ = Emotion Regulation Questionnaire; CAQ = Contrast Avoidance Questionnaire–General Emotion; IUS = Intolerance of Uncertainty Scale–Short; ASI = Anxiety Sensitivity Index–3.

**p* < .05. ***p* < .01. ****p* < .001.

**Supplemental Table 2.** *Coefficients for item-level elastic net training model*

| Item | ***β*** |
| --- | --- |
| (Intercept) | 0.0000 |
| PTCI_9_8 | 0.1492 |
| PTCI_9_9 | 0.0789 |
| PTCI_9_2 | 0.0056 |
| PTCI_9_6 | 0.0161 |
| PTCI_9_7 | 0.1156 |
| PMBS_1 | 0.1548 |
| PMBS_5 | 0.0179 |
| PMBS_8 | 0.0705 |
| PMBS_12 | 0.0788 |
| PMBS_3_r | -0.0029 |
| PMBS_15_r | -0.0002 |
| BEAQ_1 | -0.0505 |
| BEAQ_2 | 0.0035 |
| BEAQ_3 | 0.0486 |
| BEAQ_6 | 0.0267 |
| BEAQ_7 | 0.0367 |
| BEAQ_11 | -0.0047 |
| BEAQ_13 | 0.0233 |
| BEAQ_15 | 0.0069 |
| ERQ_1 | 0.0048 |
| ERQ_9 | -0.0321 |
| CAQ_GE_4 | -0.0232 |
| CAQ_GE_8 | -0.0085 |
| CAQ_GE_12 | -0.0151 |
| CAQ_GE_13 | -0.0012 |
| CAQ_GE_18 | 0.0064 |
| CAQ_GE_22 | 0.0401 |
| CAQ_GE_25 | -0.0006 |
| CAQ_GE_6 | 0.0002 |
| CAQ_GE_14 | 0.0034 |
| CAQ_GE_15 | 0.0533 |
| CAQ_GE_19 | 0.0222 |
| IUS_3 | 0.0047 |
| IUS_5 | -0.0044 |
| IUS_7 | 0.0002 |
| IUS_8 | 0.0271 |
| IUS_12 | 0.0154 |
| ASI_3_7 | 0.0495 |
| ASI_3_15 | 0.0347 |
| ASI_3_2 | 0.0661 |
| ASI_3_5 | 0.0184 |
| ASI_3_10 | 0.0194 |
| ASI_3_14 | 0.0216 |
| ASI_3_16 | 0.0282 |
| ASI_3_1 | 0.0277 |
| ASI_3_6 | 0.0066 |
| ASI_3_9 | 0.0179 |
| ASI_3_11 | 0.0037 |
| ASI_3_17 | -0.0012 |

*Note.* Items that were 0 to the fourth decimal place (rounded) were not included in this table

**Description**

We analyzed three additional elastic net models examining the relationship between risk factors and posttraumatic stress symptoms at the full scale, subscale, and item level with demographic variables included in the model. We included age as a continuous variable, and gender (Male vs. Non-Male response), sex assigned at birth (Male vs. Non-Male response), race/ethnicity (White vs. Non-White response), and sexual orientation (Heterosexual vs. Non-Heterosexual response) included as binary variables.

**Results**

Optimal penalties were selected for the full scale model (*α* = 0; *λ* = 0.09). Model fit characteristics with the training data (RMSE = 0.6920, MAE = 0.5268, *R*^2^ = 0.5209) generalized well with the testing data (RMSE = 0.6829, MAE = 0.5242, *R*^2^ = 0.5341). As can be seen, the results in term of risk factor importance were fairly similar to the findings we attained when demographics were not included, although contrast avoidance became more important than reappraisal. Optimal penalties were selected for the subscale model (*α* = 0.1; *λ* = 0.05). Model fit characteristics with the training data (RMSE = 0.6652, MAE = 0.4994, *R*^2^ = 0.5577) generalized well with the testing data (RMSE = 0.6518, MAE = 0.5033, *R*^2^ = 0.5756). Again, the results were fairly similar to our original model that did not include demographic variables. Some of the lower importance variables were not retained in the model. Optimal penalties were selected for the item-level model (*α* = 0.2; *λ* = 0.06). Model fit characteristics with the training data (RMSE = 0.6401, MAE = 0.4824, *R*^2^ = 0.5926) generalized well with the testing data (RMSE = 0.6399, MAE = 0.4908, *R*^2^ = 0.5910). Finally, the order of importance was similar to our model without demographic variables included. Variable importance for each model can be seen in supplemental figures 1-3 below.

**Supplemental Figure 1**

**
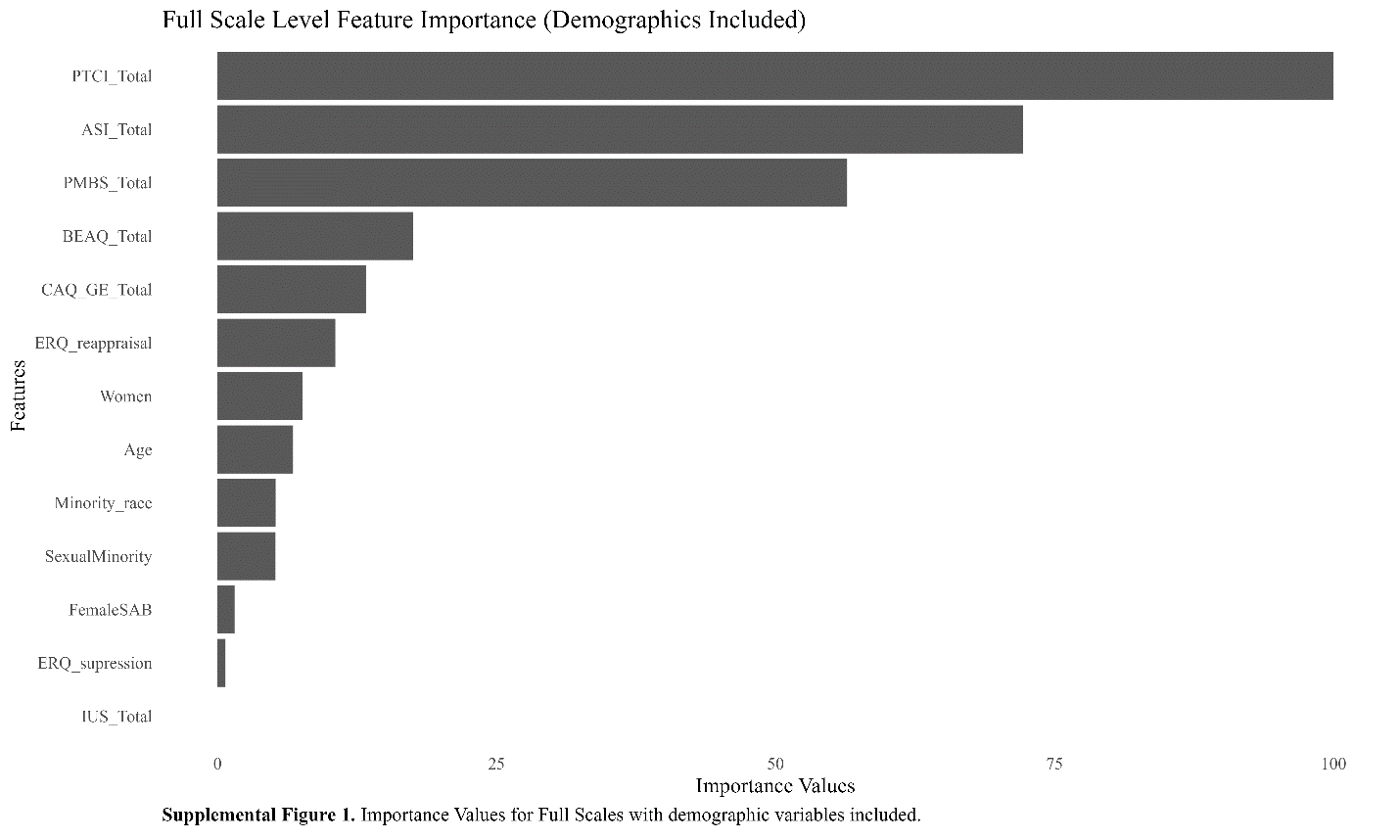
**

**Supplemental Figure 2**

**
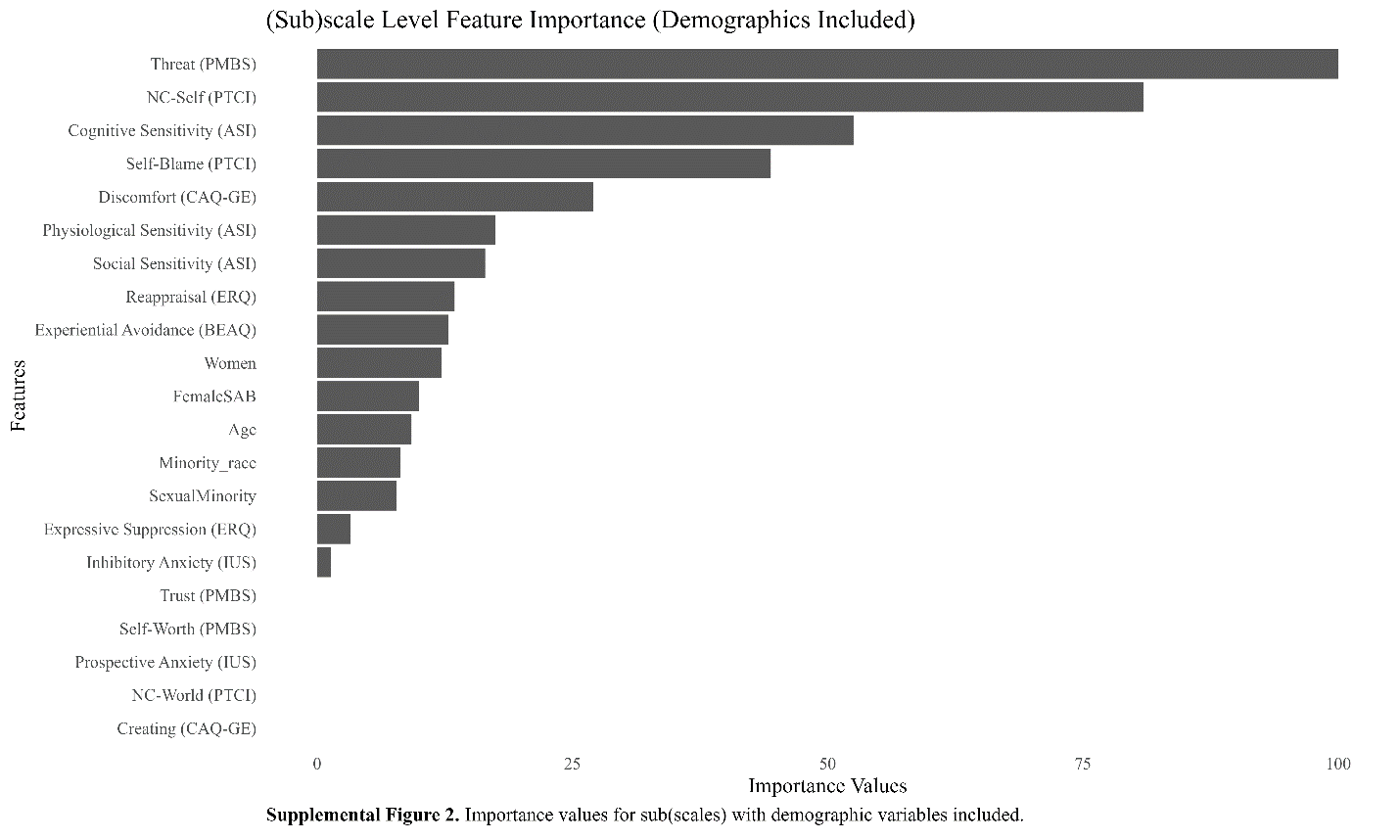
**

**Supplemental Figure 3**

**
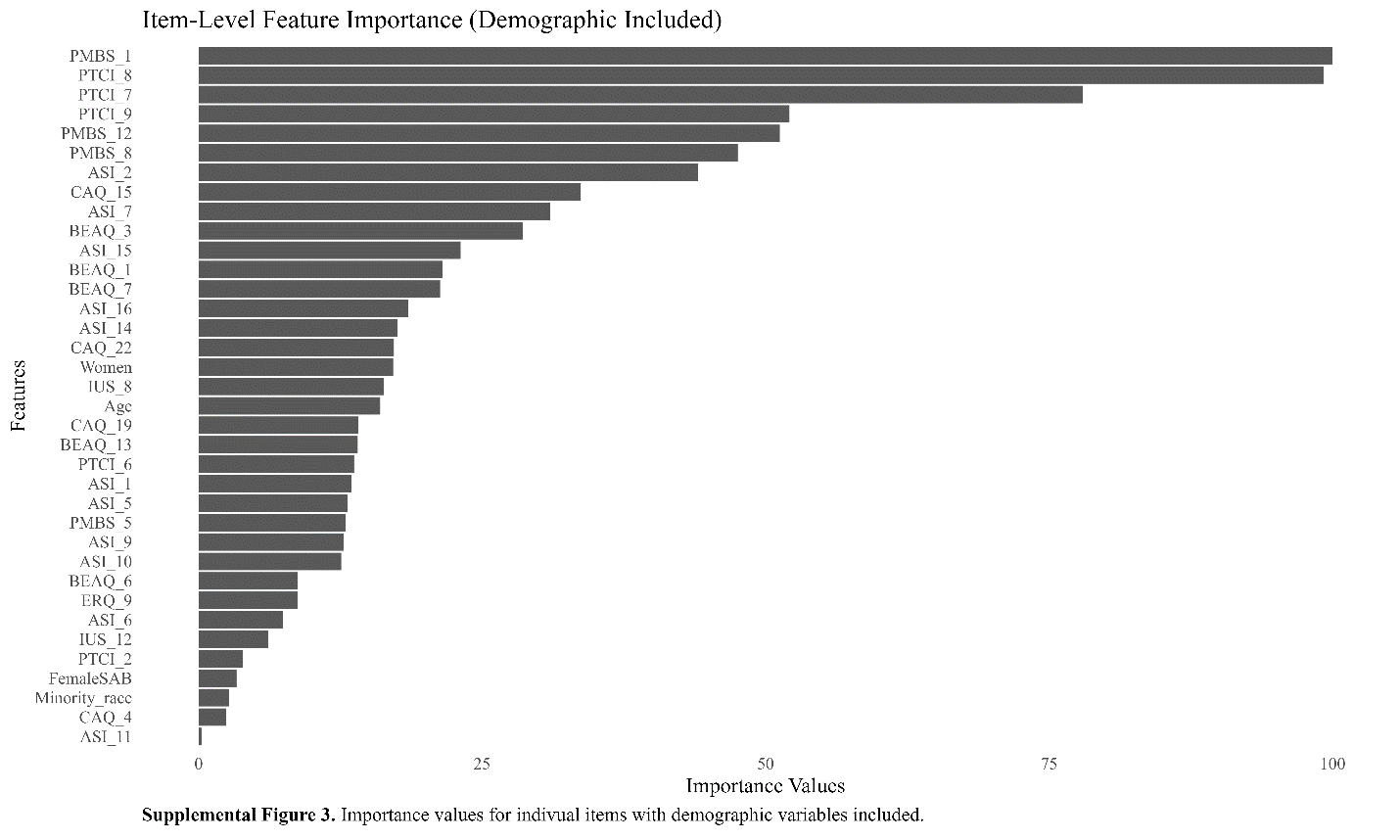
**
